# Serum Iron Levels Decreased in Patients with HBV-Related Hepatocellular Carcinoma, as a Risk Factor for the Prognosis of HBV-Related HCC

**DOI:** 10.3389/fphys.2018.00066

**Published:** 2018-02-06

**Authors:** Yanyan Wei, Wei Ye, Wei Zhao

**Affiliations:** ^1^Medical School, Southeast University, Nanjing, China; ^2^The Second Hospital of Nanjing, Medical School, Southeast University, Nanjing, China

**Keywords:** chronic hepatitis B, serum iron, liver iron overload, hepatocellular carcinoma, liver cirrhosis

## Abstract

**Background:** Hepatocellular carcinoma (HCC) is common and the second leading causes of cancer-related deaths. HCC usually occurs on the basis of chronic liver diseases. At present, the study of iron metabolism in chronic liver diseases was limited to chronic HCV infection, nonalcoholic fatty liver disease, and alcoholic liver disease. This study aimed to investigate the effect of serum iron levels on the progression of chronic HBV infection and the relationship with the prognosis of HBV-related HCC.

**Methods:** A respective study involving 277 healthy individuals as controls (HC), 295 patients with chronic hepatitis B (CHB), 224 patients with HBV-related liver cirrhosis (HBV-related LC), and 586 patients with HBV- related HCC were enrolled in this study. Hematological parameters, HBVDNA and liver biochemistry were analyzed. Child-Pugh grade and BCLC stage of the HBV-related HCC patients were calculated.

**Results:** The serum iron levels were lowest in the HBV- related HCC group as compared with HC, CHB, and HBV-related LC groups (35.07 ± 6.97, 27.37 ± 10.26, 24.53 ± 10.36 vs. 17.90 ± 0.14, *P* < 0.001). Strikingly, serum iron levels were lowest in HBV- related HCC patients with tumor size more than 10 cm as compared with HBV- related HCC patients with tumor size smaller than 3, 3–5, and 5–10 cm by subgroup analysis (22.12 ± 0.94, 21.44 ± 1.41, 15.65 ± 0.98 vs. 13.36 ± 1.15, *P* < 0.001). Serum iron levels significantly decreased with worsening Child-Pugh grades and BCLC stages in HBV-related HCC group. In addition, serum iron levels was positively correlated with Retinol-Binding Protein, total bile acid, hemoglobin, and lymphocyte and negatively correlated with white blood cell (WBC) and platelet in HBV- related HCC group. ROC curve analysis showed serum iron levels at 15.1 μmol/L as the optimal cut-off point for determining the survival of HBV-related HCC. By the Cox regression model analysis, serum iron levels <15.1 μmol/l together with higher AFP levels, worse BCLC stages, and larger tumor size showed higher mortality of HBV-related HCC patients (hazard ratio = 2.280, 95% confidence interval, 1.815–2.865; *P* < 0.001).

**Conclusions:** Serum iron levels affected the progression of chronic HBV infection. The prognosis of HBV- related HCC patients with serum iron levels <15.1 μmol/l together with higher AFP levels, worse BCLC stages, and larger tumor lesion were poor.

## Introduction

Iron is an elementary particle for life and is involved in many metabolic processes including cell growth and proliferation. However, superfluous iron in human body raises the risk of favoring neoplastic cell growth due to its capacity to take part in redox cycling and free radical production (Xiong et al., 2014). There have been some previous studies on the role of serum iron in different sites, the relationship between hemochromatosis and iron overload (Allen et al., 2008; Alakeel, 2012; Adams, 2015). Wen et al. studied a cohort of 309,443 adults in Taiwan and found that the liver and the breast were the two major cancer sites where significant cancer risks were observed for serum iron either ≥120 mu g/dL or ≥140 mu g/dL, respectively (Wen et al., 2014). Yamaguchi, K et al. illustrated that amount of free iron in the contents of enclometriotic cysts was strongly associated with greater oxidative stress and frequent DNA mutations (Yamaguchi et al., 2008). Otherwise, more previous researches focus on the relationship between liver iron overload and tumorigenesis and progression of HCC. By studying long-term survival in a cohort of 251 patients with hemochromatosis in 1996, the results showed prognosis of hemochromatosis and most of its complications, including liver cancer, depend on the amount and duration of iron excess (Niederau et al., 1996). Xiong et al. demonstrated that the effects of iron overload are associated with the tumorigenesis of lung cancer and growth of lung cancer cells in 2014 (Xiong et al., 2014). Hepatic iron accumulation was considered to be an influence factor of liver progression, and serum ferritin was also considered as a risk factor for the survival of HCC patients (Facciorusso et al., 2014). However, up to date, there are scarce of reports about the relationship between serum iron levels and the progression of chronic HBV infection, and the prognosis of HCC.

Serum iron is a routine laboratory parameter of liver biochemistry. Some previous studies focused on the role of liver iron overload in liver diseases. Nagasue et al. found the relationship between the liver iron levels and cirrhotic liver of patients with or without HCC in 1989 (Nagasue et al., 1989). Sikorska reported that increased liver iron stores could contribute to the progression of liver injury and fibrosis, and were associated with a higher risk of hepatocellular carcinoma development in 2011 (Sikorska et al., 2011). Moreover, Nirei et al. reported that the incidence of HCC could be reduced by phlebotomy treatment, which should be performed in patients with chronic hepatitis C in 2015 (Fujita and Takei, 2007). Despite the obvious role of liver iron overload increasing the risk of HCC development, the association between serum iron levels and prognosis of HBV- related HCC remains largely unknown.

In view of above-mentioned considerations, we carried out a retrospective study to illustrate the association between serum iron levels and progression of chronic HBV infection, as well as the link between serum iron levels and the prognosis of HCC.

## Methods

### Subjects

Patients admitted to the Second Hospital of Nanjing from January 2006 to August 2015 and diagnosed with CHB, HBV-related liver cirrhosis (HBV-related LC) and HBV-related HCC were recruited. The diagnosis of CHB and LC was based on the criteria established by the Chinese Medical Association (Chinese Society of Hepatology and Chinese Society of Infectious Diseases) (Hou et al., 2017). Briefly, CHB patients were defined as being positive for hepatitis B surface antigen for at least 6 months before enrolling and without evidence of cirrhosis or HCC. HBV-related LC was diagnosed based on medical history, the results of physical examination, biochemical, endoscopic and ultrasound findings, and radiological signs of cirrhosis. The diagnosis of HCC was based on the standards for the diagnosis and treatment of primary liver cancer established by the Ministry of Health of the People's Republic of China (2012). The diagnosis of HBV- related HCC was based on specific imaging findings, Hepatitis B surface antigen (HBsAg) marker positive, as well as elevated AFP ≥ 400 ng/ml sustained 1 months or AFP ≥ 200 ng/ml sustained 2 months or histological confirmation. According to criteria, a patient is considered positive for HBV- HCC if they have risk factors (HBV infection, cirrhosis) and one of the following: (i) Tumor lesion ≤ 2 cm, positive findings in at least two of four typical imaging studies [dynamic computed tomography (CT), contrast-enhanced dynamic magnetic resonance imaging (MRI), contrast-enhanced ultrasonography (CEUS), hepatic angiography], or histological confirmation. (ii) Tumor lesion >2 cm, positive findings in at least one of the above-mentioned imaging studies or histological confirmation. A positive imaging for typical HCC on dynamic CEUS, MRI, and CT is defined as arterial enhancement followed by venous washout in the delayed portal/venous phase.

Two hundred and seventy-seven healthy volunteers with normal kidney and liver function and without any history of liver diseases and history of drinking, cardiovascular, or hematological diseases, and exclusion of other acute and chronic diseases were enrolled as healthy controls.

The exclusion criteria were as follow: (i) hematological diseases and end-stage renal disease; (ii) hemorrhage of digestive tract and/ or blood transfusion 3 months prior to admission; (iii) other malignant diseases; (iv) severe chronic obstructive pulmonary disease; (v) acute events in the previous 2 weeks; (vi) coexistence of autoimmune hepatitis, drug hepatitis, alcoholic liver disease, steatohepatitis, or Wilson's disease; (vii) Other infectious diseases except hepatitis B, such as single or co-infection with hepatitis C virus hepatitis D virus, hepatitis G virus, or HIV; (viii) liver transplantation; (ix) cardiovascular diseases, transjugular intrahepatic porto systemic shunt, or portal vein thrombosis.(x) long history of smoking and drinking; (xi) malnutrition, peptic ulcer, chronic diarrhea; (xii) receiving oral contraceptive, iron supplement, immunosuppression, or Aspirin treatment; (xiii) infected with bacterium, virus, and fungus.

The study was approved by the Ethics Committee of the Second Hospital of Nanjing and written informed consent for participation was obtained. This study had no influence on the subsequent management of patients.

### Data collection

Two hundred and ninety-five patients with CHB, 224 patients with HBV-related LC, and 586 HBV-related HCC were enrolled in the present study. 277 healthy individuals were included as controls. We retrospectively reviewed the medical records of the enrolled patients. The clinical and laboratory characteristics on admission, including medical history, medication history, history of drinking and smoking, liver biochemistry, blood routine, AFP and HBV viral load were recorded.

### Statistical analysis

All the data analysis was performed using SPSS version 22.0 software (SPSS, Chicago, IL, USA). Continuous variables were expressed as mean value ± standard deviation, and categorical data were reported as percentages. The continuous data were tested using Student's *t*-test, Mann-Whitney *U*-test or one-way ANOVA. Categorical data were analyzed by the χ^2^-test. Correlation analysis between variables was conducted using Spearman's rank correlation analysis. A receiver operating characteristics (ROC) curve was constructed to estimate the optimal cut-off value of serum iron levels. Survival curves were estimated using the Kaplan-Meier method and compared using the log-rank test. Univariate analysis was performed by the Cox regression model. Multivariate analysis was carried out using the Cox proportional hazards model to generate adjusted hazard ratios and 95% confidence intervals. Any value of *P* < 0.05 was considered statistically significant.

## Results

### General subject characteristics

Two hundred and ninety-five CHB patients, 224 HBV- related LC patients, 586 HBV- related HCC patients and 277 healthy individuals were included in the present study. The demographic and clinical characteristics of the subjects are shown in Table 1. The mean age of the patients with CHB, HBV- related LC, HBV- related HCC, and HC were 40 ± 12, 42 ± 14, 47 ± 7, and 36 ± 7 years. Significant increases in serum aspartate aminotransferase (AST) and significant decreases in serum iron levels and blood platelet (PLT) were observed among four groups. However, there were no significant differences in the red blood cell (RBC), white blood cell (WBC), alanine aminotransferase (ALT), total bilirubin (TB), HBsAg, and HBVDNA levels among four groups.

**Table 1:** Baseline characteristics of patients from the entire study population.

**Variables**	**HC (*n* = 277)**	**CHB (*n* = 295)**	**HBV-related LC (*n* = 224)**	**HBV-related HCC (*n* = 586)**
Age (years)	36 ± 7	40 ± 12	42 ± 14**^a^^b^**	47 ± 7**^a^^b^^c^**
Gender (male/female)	138/139	192/103	134/90	472/114
WBC (10^9^/L)	6.37 ± 1.25	8.46 ± 1.74	6.2 ± 0.76	4.54 ± 1.93
LY (10^9^/L)	2.33 ± 0.42	1.72 ± 0.63	1.55 ± 0.61^a^^b^	1.92 ± 0.58^a^^b^^c^
RBC (10^12^/L)	4.58 ± 0.48	7.69 ± 2.92	4.13 ± 0.43^b^	4.82 ± 0.49^b^
Hb (g/L)	142 ± 16	145 ± 26	130 ± 11^b^	159 ± 22**^a^^b^**
PLT (× 10^9^/L)	212 ± 65	160 ± 63^a^	131 ± 75**^a^^b^**	136 ± 9**^a^^b^**
ALT (U/L)	24 ± 7	53 ± 12	40 ± 6	56 ± 8
AST (U/L)	20 ± 6	39 ± 7	38 ± 17	65 ± 2^a^^b^^c^
RBP (mg/L)	36.73 ± 10.22	40.01 ± 12.43^a^	30.59 ± 12.92^a^^b^	25.65 ± 7.25^a^^b^
TB (μmol/L)	17.97 ± 6.81	16.23 ± 7.47	22.06 ± 6.57	37.85 ± 10.45^a^^b^^c^
TBA (μmol/L)	3.3 ± 1.14	6.7 ± 1.45	35.32 ± 8.01	16.25 ± 2.85^a^^b^
Serum iron (μmol/L)	35.07 ± 6.97	27.37 ± 10.26	24.53 ± 10.36^a^^b^	17.90 ± 0.14^a^^b^^c^
HBsAg (log_10_IU/ml)	–	3.54 ± 3.09	3.18 ± 2.91	2.44 ± 1.53^bc^
HBVDNA (log_10_IU/ml)	–	3.38 ± 1.55	3.73 ± 0.33	4.08 ± 1.57^b^

*Data are expressed as the means ± standard deviation or n (%). P-values were calculated using one-way ANOVA, followed by Least-significant difference multiple comparisons test*.

a *P < 0.05 when compared with healthy controls*.

b *P < 0.05 when compared with chronic hepatitis B patients*.

c *P < 0.05 when compared with liver cirrhosis patients. HC, healthy controls; CHB, chronic hepatitis B; HBV-related LC, hepatitis B virus-related liver cirrhosis; HBV-related HCC, hepatitis B virus-related hepatocellular carcinoma; WBC, White blood cell; LY, Lymphocyte; RBC, Red blood cell; Hb, Hemoglobin; PLT, Platelets; ALT, Alanine-transaminase; AST, Aspartate-Aminotransferase; RBP, Retinol binding protein; TB, Total bilirubin; TBA, Total bile acid*.

The mean serum iron levels of in the HC, CHB, HBV- related LC, and HBV-related HCC groups were 35.07 ± 6.97, 27.37 ± 10.26, 24.53 ± 10.36, and 17.90 ± 0.14 μmol/l, respectively. Obviously, there were significant differences in serum iron levels among four groups (*P* = 0.023). Serum iron levels were lowest in HBV- related HCC patients. In addition, there were significant differences in serum iron levels between the CHB group and the HBV- related HCC group, as well as the patients in the HBV- related LC and HBV- related HCC groups (all of them, *P* < 0.05).

To sum up, we analyzed the association between serum iron levels with the progression of chronic HBV infection and found that serum iron levels decreased gradually with the progression of chronic HBV infection. However, whether serum iron levels could be associated with the tumor size has not been confirmed. So we studied the relationship between the serum iron levels and the tumor size of HBV- related HCC in the next step.

### Subgroup analyses of different tumor size

By subgroup analysis, 586 patients with HBV- related HCC, including 193 HBV- related HCC patients with tumor size <3 cm (32.94%), 119 HBV- related HCC patients with tumor size 3–5 cm (20.31%), 206 HBV- related HCC patients with tumor size 5–10 cm (35.15%), and 68 HBV- related HCC patients with tumor size > 10 cm (11.60%) were included as four groups, respectively. The demographic and clinical characteristics of the subjects are shown in Table 2. The mean age of the patients with HCC (<3 cm), HCC (3–5 cm), HCC (5–10 cm), and HCC (>10 cm) were 42 ± 14, 43 ± 12, 46 ± 14, and 56 ± 11 years. Significant increases in WBC, PLT, and serum AST, TB, and significant decreases in serum iron levels and retinol binding protein (RBP), were observed among four groups of patients with HBV-related HCC. Similarly, there were significant differences in Child-Pugh grade and BCLC stage of HCC patients among four groups (all of them, *P* < 0.05). However, there were no significant difference in the lymphocyte (LY), RBC, hemoglobin (Hb), ALT, TB, HBVDNA levels and cirrhosis, gender of HCC patients among four groups (all of them *P* > 0.05).

**Table 2:** Baseline characteristics of patients according to tumor size of HBV-related HCC.

**variables**	**HBV-related HCC (<3 cm) (*n* = 193)**	**HBV-related HCC (3–5 cm) (*n* = 119)**	**HBV-related HCC (5–10 cm) (*n* = 206)**	**HBV-related HCC (>10 cm) (*n* = 68)**
Age (years)	42 ± 14	43 ± 12	46 ± 14^a^^b^	56 ± 11^a^^b^^c^
Gender (male/female)	150/43	99/20	167/39	56/12
WBC (10^9^/L)	5.41 ± 0.23	4.83 ± 0.29	6.60 ± 0.38**^a^^b^**	7.09 ± 0.67**^a^^b^**
LY (10^9^/L)	1.22 ± 0.06	1.08 ± 0.07	1.23 ± 0.06	1.18 ± 0.09
RBC (10^12^/L)	4.00 ± 0.82	3.81 ± 0.92	3.96 ± 0.83	3.99 ± 1.04
Hb (g/L)	127 ± 26	123 ± 23	124 ± 22	122 ± 23
PLT (× 10^9^/L)	105 ± 5	84 ± 6	144 ± 8^a^^b^	154 ± 13^a^^b^^c^
ALT (U/L)	56 ± 6	72 ± 15	60 ± 8	68 ± 16
AST (U/L)	74 ± 9	97 ± 23	103 ± 13	151 ± 31.87^a^^b^
RBP (mg/L)	26.03 ± 12.62	25.08 ± 1.50	26.46 ± 1.26	26.03 ± 2.71
TB (μmol/L)	37.71 ± 4.92	52.32 ± 9.16	43.47 ± 7.60	53.96 ± 19.70
TBA (μmol/L)	27.07 ± 2.07	40.59 ± 5.46	31.29 ± 4.35	34.80 ± 10.28
Serum iron (μmol/L)	22.12 ± 0.94	21.44 ± 1.41	15.65 ± 0.98^a^^b^	13.36 ± 1.15^a^^b^
AFP (log_10_ng/ml)	3.20 ± 2.83	2.81 ± 2.42	4.45 ± 4.18^a^^b^	3.70 ± 3.45
HBVDNA (log_10_IU/ml)	4.12 ± 1.64	4.06 ± 1.59	4.09 ± 1.48	3.99 ± 1.29
BCLC stage (A/B/C/D)	170/8/15/0	0/74/31/14	0/92/95/19	0/17/44/7
Child-Pugh (A/B/C)	144/46/3	71/34/14	135/52/19	44/17/7

*Data are expressed as the means ± standard deviation or n (%). P-values were calculated using one-way ANOVA, followed by Least-significant difference multiple comparisons test*.

a *P < 0.05 when compared with HCC (<3 cm)*.

b *P < 0.05 when compared with HCC (3–5 cm)*.

c *P < 0.05 when compared with HCC (5–10 cm). HBV- related HCC, hepatitis B virus-related hepatocellular carcinoma; WBC, White blood cell; LY, Lymphocyte; RBC, Red blood cell; Hb, Hemoglobin; PLT, Platelets; ALT, Alanine-transaminase; AST, Aspartate-Aminotransferase; RBP, Retinol binding protein; TB, Total bilirubin; TBA, Total bile acid; AFP, Alpha-fetoprotein; BCLC stage, Barcelona Clinic Liver Cancer stage*.

We further analyzed the relationship between serum iron levels and tumor size of HBV- related HCC and found that the serum iron levels were significantly higher in the patients with HBV- related HCC (<3 cm) and HBV- related HCC (3–5 cm) than HBV- related HCC (5–10 cm) and HBV- related HCC (>10 cm) (22.12 ± 0.94, 21.44 ± 1.41, 15.65 ± 0.98 vs. 13.36 ± 1.15, *P* < 0.001), respectively. Of these, 221/586 (37.71%) HBV- related HCC patients had declined serum iron levels below the normal range. 55/586 (9.39%) HBV- related HCC patients had elevated serum iron levels beyond the normal range. Therefore, the serum iron levels in most of the HBV- related HCC patients were still within the normal range.

To sum up, it demonstrated that serum iron levels were negatively correlated with tumor size, which was lowest in tumor size more than 10 cm. However, whether serums iron levels was associated with other laboratory parameters were not yet demonstrated in HBV-related HCC subgroup and would be clarified in the next part.

### Relationship between serum iron levels and laboratory parameters in HBV-related HCC patients by subgroup analyses

**Figure 1:**
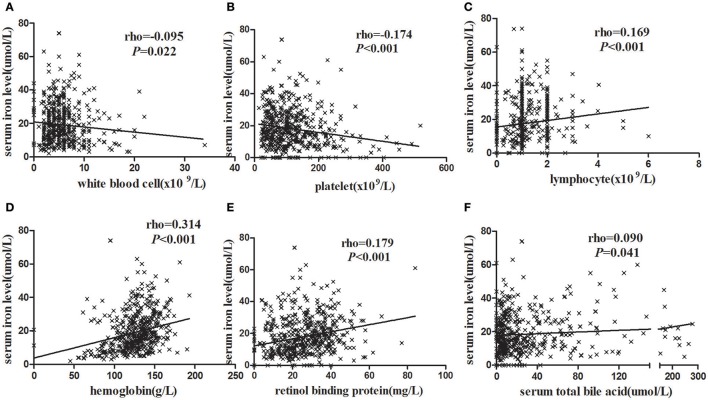
The correlation analysis between blood routine test parameters and serum iron levels. **(A)** HCC patients with higher WBC had lower serum iron levels. **(B)** HCC patients with higher LY had higher serum iron levels. **(C)** HCC patients with higher Hb had higher serum iron levels. **(D)** HCC patients with higher PLT had lower serum iron levels. **(E)** HCC patients with higher RBP were higher serum iron levels. **(F)** HCC patients with higher serum TBA were higher serum iron levels. Statistical analysis was performed with Spearman correlation analysis.

We analyzed the correlations between serum iron levels and other laboratory parameters to determine the clinical significance of decreased serum iron levels in HBV-related HCC patients. In the entire study population, Figures 1A,B showed that serum iron levels were negatively correlated with WBC counts (*r* = −0.095, *P* = 0.022) and PLT counts (*r* = −0.174 *P* < 0.001). In contrast, Figures 1C,D showed that serum iron levels were positively correlated with Hb (*r* = 0.169, *P* < 0.001) and LY (*r* = 0.314, *P* < 0.001). Similarly, Figures 1E,F showed that serum iron levels were positively correlated with serum RBP (*r* = 0.179, *P* < 0.001) and TBA (*r* = 0.090, *P* = 0.041). We also analyzed the correlation between serum iron levels and HBV DNA levels. However, there was no significant correlation between serum iron levels and HBV DNA levels (*r* = −0.030, *P* = 0.485).

To sum up, the serum iron was associated with blood routine and liver function in clinical indicators. Of these, the serum iron levels were positive correlation with Hb, LY, RBP, and TBA. In contrast, the serum iron was negative relation to the WBC and PLT counts.

### Clinical features of the HBV-related HCC patients according to the serum iron status

According to previous studies, the tumor size was an independent predictor to the prognosis of HCC (Lam et al., 2008; Yip et al., 2013; Hwang et al., 2015). It was found that the serum iron levels were negatively associated with the tumor size. However, it has not been confirmed whether the serum iron levels could impact on the overall survival rate (OS) in the patients with HBV- related HCC. In this respect, we firstly confirmed the best cut-off value of serum iron levels according to the ROC curve. Then, we further studied on the factors affecting the prognosis of HBV- related HCC.

**Figure 2:**
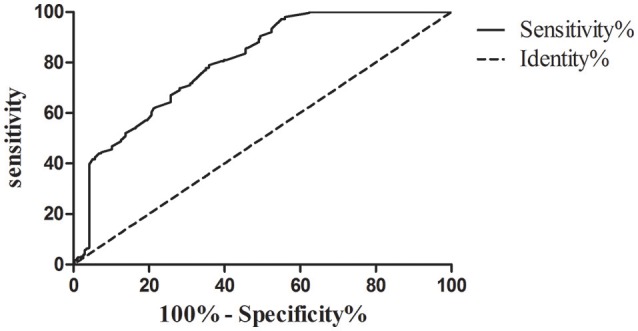
ROC curve analysis used to calculate the best cut-off point of serum iron level for differentiating the survival of patients with hepatocellular carcinoma. Best cut-off point of serum iron levels as 15.1 μmol/l (sensitivity%, 78.97% and specificity%, 64.07%; AUC [SE], 0.801 [0.018], 95% confidence limits range, 0.767–0.836, *P* < 0.0001).

The best cut-off value of serum iron levels was chosen at 15.1 μmol/l according to the ROC curve (Figure 2). The area under curve (AUC) of serum iron levels was 0.801 [95% confidence interval (95% CI): 0.767–0.836, *P* < 0.001]. According to ROC curve, we divided the patients into two groups based on their serum iron status: ≥15.1 μmol/l (*n* = 319) and <15.1 μmol/l (*n* = 267). The data showed that patients in the serum iron group more than 15.1 μmol/l had lower platelet counts (108 ± 4 vs. 134 ± 5, *P* < 0.001) than those in the serum iron levels <15.1 μmol/l group. Likewise, the data showed that the proportion of Child-Pugh A, BCLC stage A and B, HBV- related HCC (<3 cm) and HBV-related HCC (3–5 cm), and antiviral therapy before HBV- related HCC diagnosis in the serum iron more than 15.1 μmol/l group were higher than those of in the serum iron levels <15.1 μmol/l group (all of them, *P* < 0.05). However, other laboratory parameters including WBC, LY, RBC, Hb, ALT, AST, RBP, TB, TBA, AFP, and HBV viral load were no significant differences between two groups. Moreover, there were also no significant differences in patients' age, gender, and cirrhosis between two groups.

To sum up, the best cut-off point of serum iron level between HBV-related HCC survival and death was 15.1 μmol/l through the ROC curve analysis. In addition, the proportion of Child-Pugh A, BCLC stage A and B, HBV-related HCC (<3 cm) and HBV- related HCC (3–5 cm), and antiviral therapy before HBV-related HCC diagnosis in the serum iron more than 15.1 μmol/l group were higher than those of in the serum iron levels <15.1 μmol/l group. Therefore, we further studied on the risk factors associated with the survival of HBV- related HCC.

### Risk factors associated with the survival of HBV-related HCC by subgroup analyses

**Figure 3:**
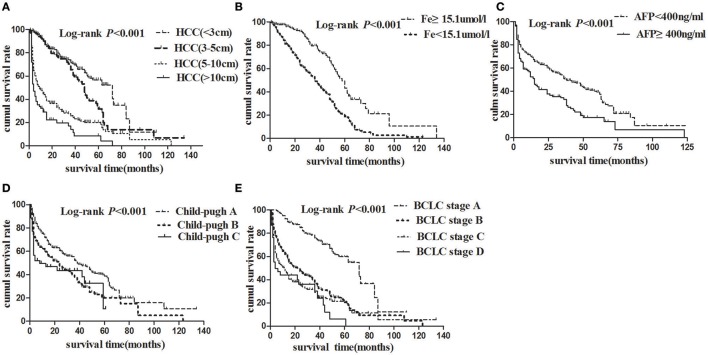
Cumulative survival plotted using the Kaplan-Meier survival curves and compared using log-rank test. **(A)** HCC patients with tumor size <3 cm (dashed line) had better overall survival than those with tumor size 3–5 cm (bold solid line), 5–10 cm (dotted line), or more than 10 cm (solid line) (*P* < 0.001). **(B)** HCC patients with higher serum iron levels (dashed line) had better overall survival than those with lower serum iron (solid line). **(C)** HCC patients with AFP < 400 ng/ml (dashed line) had better overall survival than those with AFP ≥ 400 ng/ml (dotted line). **(D)** HCC patients with Child-Pugh A (dashed line) had better overall survival than those with Child-Pugh B (dotted line), or C (solid line). **(E)** Patients with BCLC stage A (dashed line) had better overall survival than those with BCLC stage B (bold dashed line), C (dotted line), or D (solid line).

The Kaplan-Meier survival analyses were used to calculate overall survival rates among the different variables in HBV-related HCC patients. During the study follow up, 334/586 (57%) patients died. Median OS was 17 months (4–39), and 1-, 3-, and 5-years survival rate were 87.31, 59.22, and 26.15%, respectively, in the whole cohort. Figure 3A showed that there were significant differences in overall survival rates of HBV-related HCC patients with different tumor size. 1-, 3-, 5-year overall survival rate of HBV-related HCC (<3 cm), HBV-related HCC (3–5 cm), HBV- related HCC (5–10 cm), and HBV-related HCC (>10 cm) were 90.71, 90.11, 44.63, 29.06%; 71.82, 65.63, 25.67, 14.11%; and 56.62, 31.53, 20.07, 8.47%, respectively (Log-rank *P* < 0.001). Figure 3B showed that 1-year, 3-year, 5-year overall survival rate of serum iron levels more than 15.1 μmol/l and <15.1 μmol/l were 97.65, 81.29; 79.61, 49.09 and 43.28, 18.88% (Log-rank *P* < 0.001), respectively. Similarly, Figures 3C–E showed that there were significant differences in overall survival rates of HBV- related HCC patients with different serum AFP levels, Child-Pugh grades, and BCLC stages (all of them, Log-rank *P* < 0.001).

Results of univariate survival analysis demonstrated that serum iron levels were a prognostic predictor. Lower serum iron levels (<15.1 uml/l) was associated with worse overall survival rate (hazard ratio (HR) = 2.578, *P* < 0.001). Other prognostic variables were presented in Table 3. Of these, tumor size <3 cm (HR = 0.168, *P* < 0.001), tumor size 3–5 cm (HR = 0. 216, *P* < 0.001), tumor size 5–10 cm (HR = 0.640, *P* = 0.004), BCLC stage A (HR = 0.287, *P* < 0.001), antiviral therapy (ART) before HBV-related HCC diagnosis (HR = 0.443, *P* < 0.001), ART after HBV-elated HCC diagnosis (HR = 0.529, *P* < 0.001), and AFP < 400 ng/ml (HR = 0.570, *P* < 0.001) were related to better overall survival rate.

**Table 3:** Demographic and clinical characteristics of patients according to serum iron levels.

**Variables**	**Serum iron levels (<15.1 μmol/l) (*n* = 267)**	**Serum iron levels (≥15.1 μmol/l) (*n* = 319)**	***P*-value**
Age (years)	56 ± 11	56 ± 10	0.990
Gender			0.289
Male	210 (78.65%)	262 (82.13%)	
Female	57 (21.35%)	57 (17.87%)	
WBC (10^9^/L)	6.16 ± 0.24	5.72 ± 0.18	0.141
LY (10^9^/L)	1.15 ± 0.04	1.20 ± 0.04	0.401
RBC (10^12^/L)	3.87 ± 0.99	4.01 ± 0.79	0.297
Hb (g/L)	122 ± 24	127 ± 24	0.406
PLT (10^9^/L)	134 ± 5	108 ± 4^b^	*<0.001*
ALT (U/L)	50.45 ± 4.42	62.17 ± 6.06	0.132
AST (U/L)	93.86 ± 8.42	95.55 ± 9.29	0.894
RBP (mg/L)	25.43 ± 12.85	26.70 ± 12.79^b^	0.230
TB (μmol/L)	41.80 ± 4.87	41.88 ± 3.74	0.990
TBA (μmol/L)	31.57 ± 2.87	33.25 ± 2.60	0.665
HBVDNA (log_10_IU/ml)	4.20 ± 1.59	4.04 ± 0.085	0.163
AFP (ng/ml)			0.126
<400 ng/ml	200 (74.91%)	255 (79.94%)	
≥400 ng/ml	67 (25.09%)	63 (19.75%)	
BCLC stage			*<0.001*
A	52 (19.48%)	118 (36.99%)	
B	92 (34.46%)	99 (31.03%)	
C	96 (35.96%)	89 (27.90%)	
D	27 (10.11%)	13 (4.08%)	
Child-Pugh grade			*0.016*
A	168 (62.92%)	226 (70.85%)	
B	71 (26.49%)	78 (24.45%)	
C	28 (10.49%)	15 (4.70%)	
Cirrhosis			0.917
No	41 (15.36%)	48 (15.05%)	
yes	226 (84.64%)	271 (84.95%)	
Antiviral therapy timing			0.006
before HCC diagnosis	115 (43.07%)	161 (50.47%)	
after HCC diagnosis	48 (17.98%)	73 (22.88%)	
non-antiviral therapy	104 (38.95%)	85 (26.65%)	
HCC tumor size			*<0.001*
HCC (<3 cm)	62 (23.22%)	131 (41.07%)	
HCC (3–5 cm)	46 (17.23%)	73 (22.88%)	
HCC (5–10 cm)	115 (43.07%)	91 (28.53%)	
HCC (>10 cm)	44 (16.48%)	24 (7.52%)	

*Data are expressed as the means ± standard deviation or n (%). P-values were calculated using independent-samples T-test for continuous variables and χ^2^-test for categorical data. WBC, White blood cell; LY, Lymphocyte; RBC, Red blood cell; Hb, Hemoglobin; PLT, Platelets; ALT, Alanine-transaminase; AST, Aspartate-Aminotransferase; RBP, Retinol binding protein; TB, Total bilirubin; TBA, Total bile acid; AFP, Alpha-fetoprotein; BCLC stage, Barcelona Clinic Liver Cancer stage; HCC, hepatitis B virus-related hepatocellular carcinoma. The values in italics demonstrated that P-values were <0.05 and considered to be statistically significant*.

In order to eliminate the confounding factors, Cox proportional hazards model was used to evaluate the risk factors for the survival of HBV- related HCC. Variables included in the analysis were serum iron levels, AFP levels, HBV- related HCC tumor size, BCLC stages, and antiviral therapy timing. Table 4 showed that serum iron levels (HR = 2.280, *P* < 0.001) were independent risk factors of the survival of HBV-related HCC. In contrast, lower AFP levels and smaller tumor size, better BCLC stage were independent protective factors for the survival of HBV-related HCC. Namely, the serum iron levels <15.1 μmol/l was a significant risk factor for HBV- related HCC development with a hazard ratio of 2.28 (95%CI, 1.82–2.87; *P* < 0.001) together with higher AFP levels, larger tumor size and worse BCLC stages.

**Table 4:** Results of univariate and multivariate analyses in 586 HCC patients.

**Variables**	**HR**	**95%CI**		***P*-value**	**HR**	**95%CI**		***P-value***
		**Lower**	**Upper**			**Lower**	**Upper**	
Age	0.999	0.990	1.009	0.872				
Gender (male vs. female)	0.971	0.741	1.271	0.830				
Serum iron levels <15.1μmol/l (vs. ≥15.1 μmol/l)	2.578	2.061	3.226	*<0.001*	*2.280*	*1.815*	*2.865*	*<0.001*
AFP levels (<400 ng/ml vs. ≥400 ng/ml)	0.570	0.448	0.714	<0.001	0.749	0.585	0.960	*0.022*
HCC (<3 cm) (vs. HCC > 10 cm)	0.168	0.118	0.239	*<0.001*	0.602	0.347	1.047	*0.023*
HCC (3–5 cm) (vs. HCC >10 cm)	0.216	0.144	0.322	*<0.001*	0.235	0.155	0.358	*<0.001*
HCC (5–10 cm) (vs. HCC > 10 cm)	0.640	0.473	0.865	*0.004*	0.632	0.466	0.856	*0.003*
Child-Pugh A (vs. C)	0.673	0.448	1.012	0.057				
Child-Pugh B (vs. C)	0.892	0.576	1.380	0.607				
BCLC stage A (vs. D)	0.287	0.177	0.466	*<0.001*	0.340	0.168	0.687	*0.003*
BCLC stage B (vs. D)	0.989	0.638	1.532	0.960				
BCLC stage C (vs. D)	1.158	0.748	1.792	0.511				
ART before HCC diagnosis (vs. non-ART)	0.443	0.349	0.563	<0.001				
ART after HCC diagnosis (vs. non- ART)	0.529	0.389	0.718	<0.001				
Cirrhosis (no vs. yes)	1.301	0.987	1.729	0.070				

*Univariate analysis was performed by the Cox regression model. Multivariate analysis was performed by the Cox proportional hazard model using an enter procedure. HR, hazard ratio; CI, confidence interval; HCC, hepatitis B virus-related hepatocellular carcinoma; BCLC stage, Barcelona Clinic Liver Cancer stage; AFP, Alpha-fetoprotein; ART, Antiviral therapy. The values in italics demonstrated that P-values were <0.05 and considered to be statistically significant*.

## Discussion

Hepatocellular carcinoma (HCC) is known to have a poor prognosis. The pathogenesis of HCC has been reported by many research groups in the worldwide. Of these, several reports indicated that liver iron overload might be involved in the pathogenesis of HCC (Nagasue et al., 1989; Hajiani et al., 2005; Fujita and Takei, 2007; Lefkowitch, 2008). Moreover, Fargion et al. have demonstrated that iron could facilitate persistent hepatitis B or C infection and act as a co-factor in the pathogenesis of HCC (Fargion et al., 1991). However, there are scarce of reports about the relationship between serum iron levels and the progression of chronic HBV infection, as well as the relationship between serum iron levels and the prognosis of HBV-related HCC. Therefore, firstly, we compared the differences of serum iron levels among different chronic HBV infected stages. It indicated that the serum iron levels were lowest in HBV-related HCC patients as compared with CHB and HBV-related LC patients. Secondly, by subgroup analysis, it demonstrated that the serum iron levels were negatively correlated with the tumor size. Thirdly, it was found that the best cut-off value of serum iron levels was chosen at 15.1 μmol/l by ROC curve analysis. Finally, by Cox proportional hazards model analysis, it was found that the serum iron levels <15.1 μmol/l was a significant risk factor for the survival of HBV- related HCC (HR = 2.28, 95%CI, 1.82–2.87; *P* < 0.001) together with higher AFP levels, larger tumor size and worse BCLC stages.

Iron is an essential component in DNA synthesis and in respiratory and oxidative metabolism (Sussman, 1992). Iron is considered a putative element that interacts with oxygen radicals in inducing liver damage and fibrosis (Sumida et al., 2009; Sikorska et al., 2016). Clinical correlations have been made linking cellular iron content to the development of cancer in humans. Iron reduction therapy may be promising in chronic liver disease patients to reduce serum transaminase activities (Sussman, 1992). By analyzing the demographic and clinical characteristics of CHB, HBV-related LC, HBV-related HCC, and healthy controls groups, it was indicated that significant increases in serum AST levels and significant decreases in serum iron levels and blood platelet in patients with HBV-related HCC. Therefore, the abnormal accumulation of serum iron leads to a change in the condition of the liver disease, which is part of the disease process. To sum up, these results were similarly with previous studies about iron interact with oxygen radicals in inducing liver damage and fibrosis (Smith et al., 1993; Sumida et al., 2009; Alakeel, 2012; Guo et al., 2012).

As mentioned, serum iron levels were associated with progression of chronic HBV infection in our study. So far, most of studied focused on relationship between the liver iron overload and HBV-related HCC in patients with HCV infection, NAFLD or AFLD (Mahmood et al., 2004; Nahon et al., 2008; Mueller et al., 2009; Starley et al., 2010; Nirei et al., 2015). There are rarely reported about the relationship between the serum iron levels and tumor size, as well as the relationship between serum iron levels and the prognosis of HBV-related HCC. In this regards, by subgroup analysis, serum iron levels was significantly lower in HBV- related HCC patients with tumor size more than 10 cm as compared with HBV- related HCC patients with tumor size smaller than 3, 3–5, and 5–10 cm. Meanwhile, significant increases in serum AST levels, TB levels, WBC and PLT counts, and significant decreases in RBP levels, were observed among four groups classified by tumor size. In general, 37.7% HBV- related HCC patients had declined serum iron values below the normal range, but the serum iron levels in most of the HBV- related HCC patients were still within the normal range. To sum up, serum iron levels were negatively associated with tumor size. However, serum iron levels in most of the HBV- related HCC patients were still within the normal range, or even higher. Therefore, functional iron may be low or normal in peripheral blood, but tissue iron might be high. This result was similarly with previous studies in HCV infection (Mahmood et al., 2004; Asia-Pacific Working Party on Prevention of Hepatocellular Carcinoma, 2010). In addition, Sorafenib combined with Deferasirox could enhance Sorafenib-induced apoptosis in HCC have been reported (Urano et al., 2016). Therefore, iron chelation could be novel adjuvant therapy for HCC.

Besides the above analysis of serum iron were negatively related to HBV- related HCC tumor size in our study, we further studied on the relationship between serum iron levels and the prognosis of HBV- related HCC. There were some previous reports on liver iron overload as an influencing factor contributing to the progression of HCC (Fargion et al., 1991; Chapoutot et al., 2000; Furutani et al., 2006; Nahon et al., 2008; Chen and Chloupkova, 2009; Sorrentino et al., 2009). However, the impact of serum iron levels on the survival of HBV- related HCC patients was poorly reported. Firstly, by ROC curve, it was found that the best cut-off value of serum iron levels was chosen at 15.1 μmol/l. Then, it showed that 1-, 3-, 5-year overall survival rate of serum iron more than 15.1 and <15.1 μmol/l were 97.65, 81.29; 79.61, 49.09, and 43.28, 18.88%(Log-rank *P* < 0.001) by the Kaplan-Meier survival analyses. In addition, by univariate analysis, it demonstrated that serum iron levels were a prognostic predictor. Lower serum iron levels (<15.1 uml/l) was associated with worse overall survival rate (HR = 2.578, *P* < 0.001). Other prognostic variables included AFP levels, HCC tumor size, BCLC stage, and ART timing. By Cox proportional hazards model, we found that serum iron levels (HR = 2.280, *P* < 0.001) was an independent risk factors for the survival of HBV- related HCC. In contrast, lower AFP levels, smaller tumor size, and better BCLC stage were independently protective factors for the survival of HBV- related HCC. Briefly, the serum iron levels <15.1 μmol/l was a significant risk factor for the survival of HBV- related HCC (HR = 2.28, 95% CI, 1.82–2.87; *P* < 0.001) together with higher AFP levels, larger tumor size and worse BCLC stages. Although previous studies have demonstrated that AFP levels (Matsumoto et al., 2003; Ma et al., 2013; Ladep et al., 2015), tumor size (Yip et al., 2013), and BCLC stage (Fernandez-Ruiz et al., 2009; Guo et al., 2017) were independent predictors for the survival of HCC. There were firstly reported that the serum iron levels <15.1 μmol/l was a significant risk factor for the survival of HBV- related HCC.

There were some reports about the correlation between hemoglobin levels and serum iron levels in different patients with chronic hepatitis (Wang et al., 2013; Bai et al., 2014; Nirei et al., 2015; Park et al., 2015). However, there were no reports about the correlation between serum iron levels and liver function indicators or blood routine test exception hemoglobin for HCC patients. In our study, we found that serum iron levels were positively correlated with Hb levels. Furthermore, the data suggested that serum iron levels was also positively correlated with RBP, TBA levels and LY counts, and negatively correlated with WBC and PLT counts. However, no significant correlation between serum iron and HBV DNA levels was found.

## Conclusion

These observations suggested the correlations between serum iron levels and progression of chronic HBV infection, especially for the negative correlation between serum iron levels and HBV- related HCC tumor size. In patients with HBV-related HCC, the lowest serum iron levels were found in patients with tumor size more than 10 cm by subgroup analysis. HBV- related HCC patients with serum iron levels lower than 15.1 μmol/l was an independent risk factor together with higher AFP levels, larger tumor size, and worse BCLC stages for the survival of HCC.

Because of serum iron was a non-invasive laboratory test compared with tissue iron levels. Therefore, the major highlights of our study were the novelty of our results that provides a dependable, repeatable and easily measurable prognostic factor for HBV- related HCC patients. Furthermore, a cut-off point of serum iron levels was suggested, thus enabling clinicians to optimize the use of serum iron as a prognostic marker in daily practice. It may also shed a new light on the development of biomarkers for HBV-related disease surveillance and be also used as an auxiliary indicator to judge the prognosis of HBV- related HCC patients. However, our paper has some limitations. Firstly, although only patients with chronic HBV infection were enrolled in our study could reduce the biases for interpretation of the results, the number of patients requires further validation by larger multicenter and prospective studies. Secondly, there were scarce of results of blood tests in iron metabolism. Hence, the relationship between serum iron and iron metabolism was not provided in the current study. As such, we will further study the relationship between serum iron and iron metabolism series.

## Author contributions

YW researched the data, contributed to the discussion, wrote the manuscript, reviewed and edited the manuscript. WY and WZ researched the data, contributed to the discussion, reviewed and edited the manuscript. YW is the guarantor of this work and, as such, had full access to all the data in the study and takes responsibility for the integrity of the data and the accuracy of the data analysis.

### Conflict of interest statement

The authors declare that the research was conducted in the absence of any commercial or financial relationships that could be construed as a potential conflict of interest.
